# The SleepFit Tablet Application for Home-Based Clinical Data Collection in Parkinson Disease: User-Centric Development and Usability Study

**DOI:** 10.2196/16304

**Published:** 2021-06-08

**Authors:** Alessandro Mascheroni, Eun Kyoung Choe, Yuhan Luo, Michele Marazza, Clara Ferlito, Serena Caverzasio, Francesco Mezzanotte, Alain Kaelin-Lang, Francesca Faraci, Alessandro Puiatti, Pietro Luca Ratti

**Affiliations:** 1 Institute of Information Systems and Networking Department of Innovative Technologies University of Applied Sciences and Arts of Southern Switzerland Lugano Switzerland; 2 College of Information Studies University of Maryland College Park, MD United States; 3 Information & Communication Technology Ente Ospedaliero Cantonale Bellinzona Switzerland; 4 Neurocenter of Southern Switzerland Lugano Switzerland; 5 Faculty of Biomedical Sciences University of Southern Switzerland Lugano Switzerland; 6 Medical School University of Bern Bern Switzerland

**Keywords:** Parkinson disease, ecological momentary assessment, finger-tapping test, subjective scales, sleep diaries, tablet application, home-based system

## Abstract

**Background:**

Parkinson disease (PD) is a common, multifaceted neurodegenerative disorder profoundly impacting patients' autonomy and quality of life. Assessment in real-life conditions of subjective symptoms and objective metrics of mobility and nonmotor symptoms such as sleep disturbance is strongly advocated. This information would critically guide the adaptation of antiparkinsonian medications and nonpharmacological interventions. Moreover, since the spread of the COVID-19 pandemic, health care practices are being reshaped toward a more home-based care. New technologies could play a pivotal role in this new approach to clinical care. Nevertheless, devices and information technology tools might be unhandy for PD patients, thus dramatically limiting their widespread employment.

**Objective:**

The goals of the research were development and usability evaluation of an application, SleepFit, for ecological momentary assessment of objective and subjective clinical metrics at PD patients’ homes, and as a remote tool for researchers to monitor patients and integrate and manage data.

**Methods:**

An iterative and user-centric strategy was employed for the development of SleepFit. The core structure of SleepFit consists of (1) an electronic finger-tapping test; (2) motor, sleepiness, and emotional subjective scales; and (3) a sleep diary. Applicable design, ergonomic, and navigation principles have been applied while tailoring the application to the specific patient population. Three progressively enhanced versions of the application (alpha, v1.0, v2.0) were tested by a total of 56 patients with PD who were asked to perform multiple home assessments 4 times per day for 2 weeks. Patient compliance was calculated as the proportion of completed tasks out of the total number of expected tasks. Satisfaction on the latest version (v2.0) was evaluated as potential willingness to use SleepFit again after the end of the study.

**Results:**

From alpha to v1.0, SleepFit was improved in graphics, ergonomics, and navigation, with automated flows guiding the patients in performing tasks throughout the 24 hours, and real-time data collection and consultation were made possible thanks to a remote web portal. In v2.0, the kiosk-mode feature restricts the use of the tablet to the SleepFit application only, thus preventing users from accidentally exiting the application. A total of 52 (4 dropouts) patients were included in the analyses. Overall compliance (all versions) was 88.89% (5707/6420). SleepFit was progressively enhanced and compliance increased from 87.86% (2070/2356) to 89.92% (2899/3224; *P*=.04). Among the patients who used v2.0, 96% (25/26) declared they would use SleepFit again.

**Conclusions:**

SleepFit can be considered a state-of-the-art home-based system that increases compliance in PD patients, ensures high-quality data collection, and works as a handy tool for remote monitoring and data management in clinical research. Thanks to its user-friendliness and modular structure, it could be employed in other clinical studies with minimum adaptation efforts.

**Trial Registration:**

ClinicalTrials.gov NCT02723396; https://clinicaltrials.gov/ct2/show/NCT02723396

## Introduction

Parkinson disease (PD) is a neurodegenerative disorder affecting 1.5% of subjects aged older than 60 years [1]. Its prevalence in the general population is estimated between 1/10,000 and 4/1000 [2]. A progressively impaired motor function leads to loss of autonomy in daily living and reduced quality of life of PD patients [3]. Besides motor symptoms, PD also features nonmotor symptoms involving, among others, cognition, emotional state, autonomic functions, and sleep. From 65% to 95% of PD patients report disturbed sleep or daytime sleepiness [4,5], which further impairs their quality of life or the quality of life of their families [6,7]. Moreover, a substantial proportion of patients with PD report prominent spontaneous, transitory improvements in mobility after sleep and before taking the first morning dose of dopaminergic medications. This phenomenon is referred to as “sleep benefit” [8].

To characterize the wide variations of motor and nonmotor symptoms within the same day and their day-to-day variability in individual patients, multiple repeated assessments are necessary. Moreover, prospective assessment of subjective symptoms is essential for the collection of data that is unbiased by patients' recall. This issue can be critical in patients with PD, who may have subclinical cognitive dysfunction [9]. In fact, a sizeable proportion of patients tend to over or underestimate their symptom severity in retrospect at hospital consultations [10]. Tracking subjective symptoms and their variation is crucial for clinical follow-up of patients with PD. Information derived from patients’ subjective perceptions is as important as objective motor features to optimally adapt pharmacological and nonpharmacological treatments [11].

In addition, remote monitoring has very recently become a strong and urgent need for clinical care since many national health authorities have encouraged patients with chronic diseases not to attend their follow-up consultations unless seeking urgent care in the attempt to limit the spread of the COVID-19 pandemic.

Ecological momentary assessment is a technique involving repeated, prospective sampling of subjects’ current behaviors or experiences in real time in his or her natural environment. Ecological momentary assessment aims to study various phenomena in real-world contexts, minimize recall bias, and maximize ecological validity [12].

This approach has been applied to collect both subjective and objective data from patients with PD in only two preliminary studies, to the best of our knowledge. In the study by van Gilst et al [13], objective metrics of motor performance were collected using an electronic test, while a self-administered pen-and-paper motor diary was used to record subjective symptoms. In a feasibility study by Bot et al [14], only objective metrics of motor features were prospectively collected.

To ensure that patient symptoms are collected prospectively, self-assessed information needs to be recorded at specific time points during the day and integrated with objective assessment in a holistic way. Electronic and information technology devices are thus essential for ecological momentary assessment. However, digital technologies can be impractical for PD subjects, who often have impaired hand dexterity.

Although software apps for smartphones exist for PD [15,16], their use can be challenging for the patients, mainly because of the small screen size on these devices. We are not aware of any tablet-based applications designed for patients with PD that take into account the ergonomic issues specific to this population.

Several systems have been developed to date to collect information on mobility and motor symptoms directly in the home of patients with PD [14-20]. These home-based systems are designed to collect objective [14-20] or subjective [14,19] metrics of motor features. However, to the best of our knowledge, there are currently no available software applications that prospectively record both objective and subjective metrics of motor and nonmotor features of PD.

To meet the need for a tool capable of collecting both objective and subjective metrics of motor and nonmotor symptoms that was handy for PD patients, our group developed an application for tablets called SleepFit. This new application, designed specifically for patients with PD, combines subjective motor scales, a sleepiness scale, and a sleep diary. SleepFit proposes questions and tasks to the patients at specific time points during the day and attributes an exact timestamp indicating when the data are collected. The data collected with SleepFit are automatically stored in a remote server and can be retrieved, integrated with data from other sources, and managed by means of a web application, the SleepFit Researcher Portal.

We aim here to introduce this home-based monitoring system and the improvements that were made in v2.0, the most recent version discussed in the paper. In addition, we outline specific requirements for software applications targeted to patients with PD. We then share the lessons learned through an iterative development centered on the real needs of patients with PD in real-life conditions. Finally, we evaluate patient compliance and satisfaction with this new tool.

## Methods

### Participants

This study was conducted on the first 56 consecutive participants enrolled in the Sleep & Move study between March 2016 and December 2018. Participation in the study was proposed to all consecutive patients meeting the eligibility criteria who were attending the outpatient department of the Movement Disorder Unit of the Neurocenter of Southern Switzerland in Lugano, Switzerland. Other patients volunteered to participate after advertisement of the study in the magazine of the Swiss Parkinson patients’ association and in public conferences organized by the same association. Eligibility criteria were mild to moderate idiopathic PD (no atypical parkinsonism) [21] (Hoehn & Yahr stage >1 and ≤3) [22], no cognitive impairment (Mini-Mental State Examination score ≥26/30) [23], no active depression (Beck Depression Inventory score <14/63) [24], no deep brain stimulation.

The initial visit was performed at the hospital by a senior neurologist (PLR) expert in sleep medicine and movement disorders. Each patient underwent a full general clinical and neurological examination including the Movement Disorders Society Unified Parkinson’s Disease Rating Scale with the motor part (III) performed during the “on” phase in patients with motor fluctuations. During the initial visit, each patient was given instructions for the use of the SleepFit application. The 14-day home period started at the end of the initial visit for each patient. A follow-up visit was performed at the hospital at day 14 by the same person. The satisfaction questionnaires were collected during this follow-up visit. No economic compensation was provided to the participants. Each participant was offered a reimbursement of the travel expenses for the two visits.

### Sleep & Move Study

**Figure 1:**
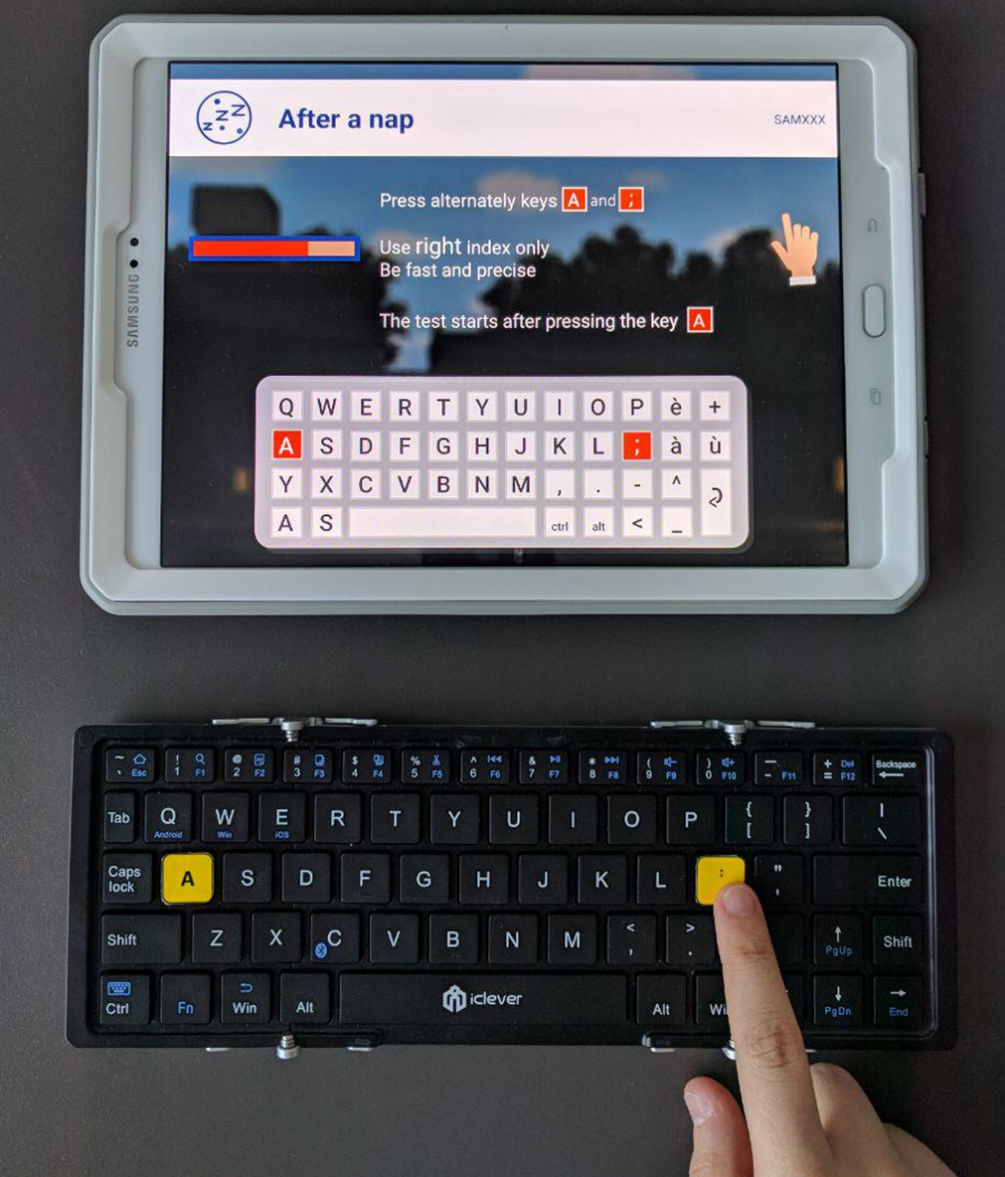
The SleepFit home-based system with application installed on a tablet (top) and portable wireless keyboard used to perform motor task (eg, after napping; bottom).

This usability study was conducted in the framework of the Sleep & Move study [25], which focused on the characterization of sleep benefit in PD. A second objective of Sleep & Move was to test the usability and acceptability of the SleepFit application by patients with PD and add improvements based on patient experience and feedback. Participation in this study was on a voluntary basis. The study was conducted in accordance with the Declaration of Helsinki and written consent was provided by all participants. The study was reviewed and approved by the Ethical Committee of the Canton of Ticino (PB_2016-00056) and registered at ClinicalTrials.gov [NCT02723396] before recruitment began. All participants were asked to use the SleepFit home-based system for 14 consecutive days. The system is composed of an Android tablet on which the SleepFit application is installed and a portable wireless keyboard used for a specific motor task (Figure 1).

### Sessions

Four sessions daily were to be completed by the patients: the “on waking” session was scheduled 30 minutes after waking up in the morning; “after medications”, 1 hour after the intake of the first dose of dopaminergic medications in the morning; “afternoon”, in the afternoon before taking dopaminergic medication (where applicable); and finally, “evening”, just before bedtime.

The timing of these 4 daily sessions was chosen considering several disease-related and practical considerations. Four time points a day is the optimum to reliably characterize the daily profile of fluctuation of PD motor or nonmotor symptoms without excessively interfering with the patients’ daily routines. Moreover, these 4 moments of the day are the most adapted, from a clinical standpoint, to seize both spontaneous fluctuations of PD and the ones related to dopaminergic medication intake and end-of-dose effects. This format is also in accordance with routine clinical practice for patients in movement disorder units. An additional session (“nap”) could be performed after any nap taken by the patient during the day at his or her request.

### Specific Tasks

**Figure 2:**
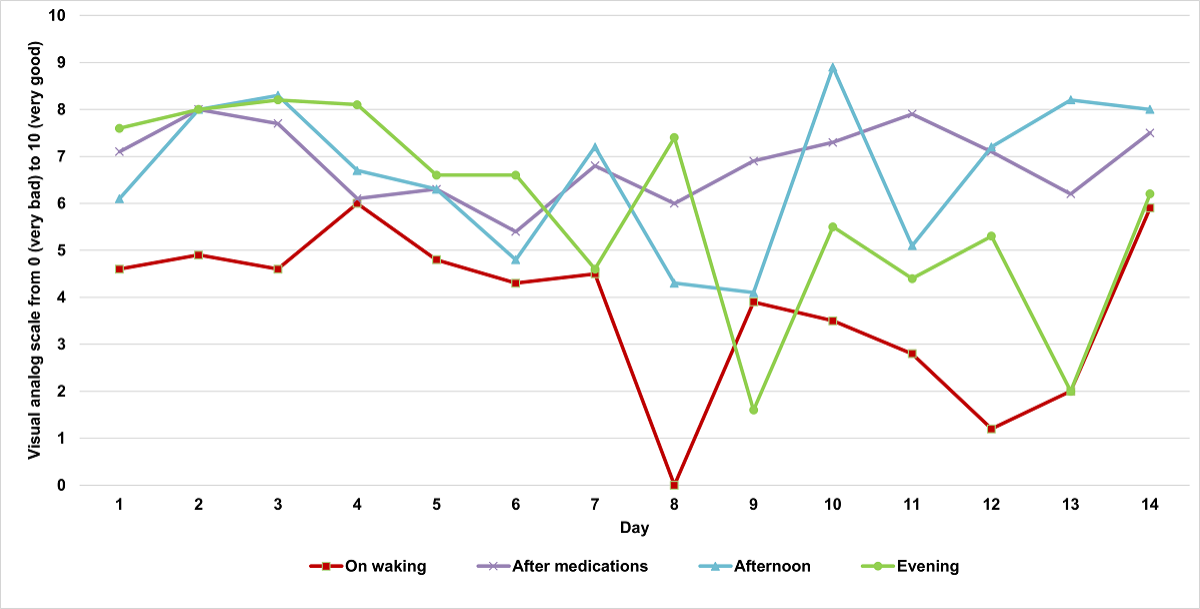
Answers to “How would you rate your capability to move right now?” collected on SleepFit for a single patient.

In each session, the patient was asked to perform 2 sets of tasks: subjective scales and a motor test. Subjective scales measure motor status, sleepiness, and emotional state. They include questions 1, 2, 3, and 4 of the Scales for Outcome in Parkinson’s Disease Diary Card [26], a visual analog scale (score 0 to 10) [10,27] for perceived overall mobility, tremor, mood, and anxiety level (one scale for each), and the Stanford sleepiness scale [28]. An example of the 14-day patient answer evolution is shown in Figure 2.

**Figure 3:**
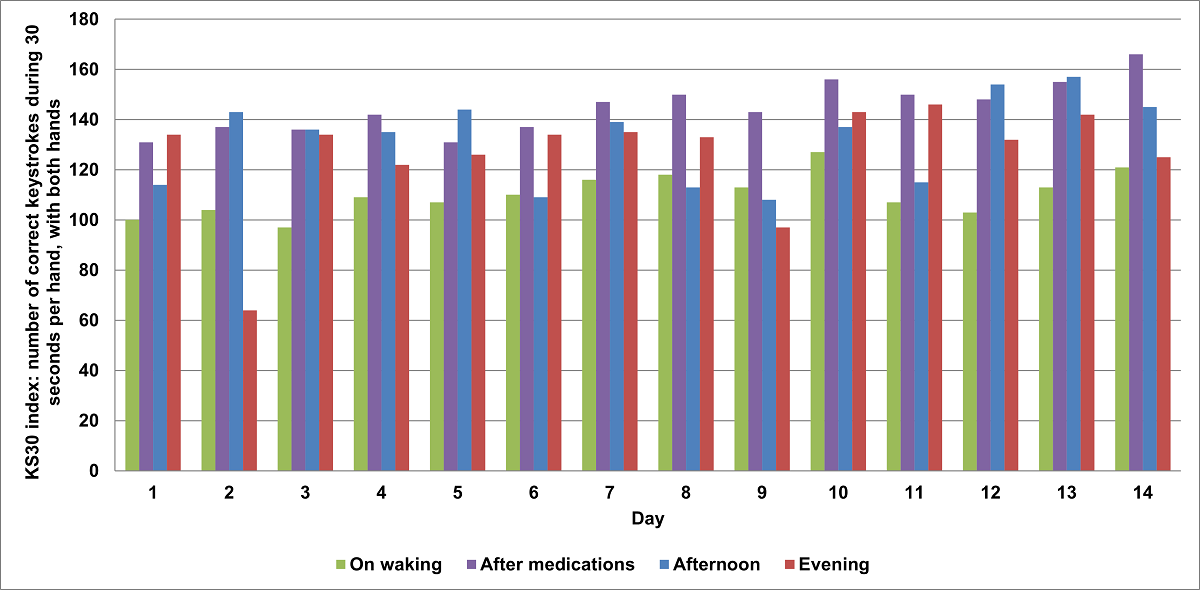
Objective data collected during the execution of a Fit Test by a single patient.

The motor test consists in a digital finger-tapping test that we named the Fit Test, which is based on the previously published Bradykinesia-Akinesia Incoordination test [29,30]. In this test, the subject is asked to strike 2 keys on an external keyboard at 10 cm from each other repeatedly and alternatively for 30 seconds using one hand at a time (Figure 1). We computed the same parameters validated in Noyce et al [29] (see Figure 3 for an example of a parameter computed for a single subject).

**Figure 4:**
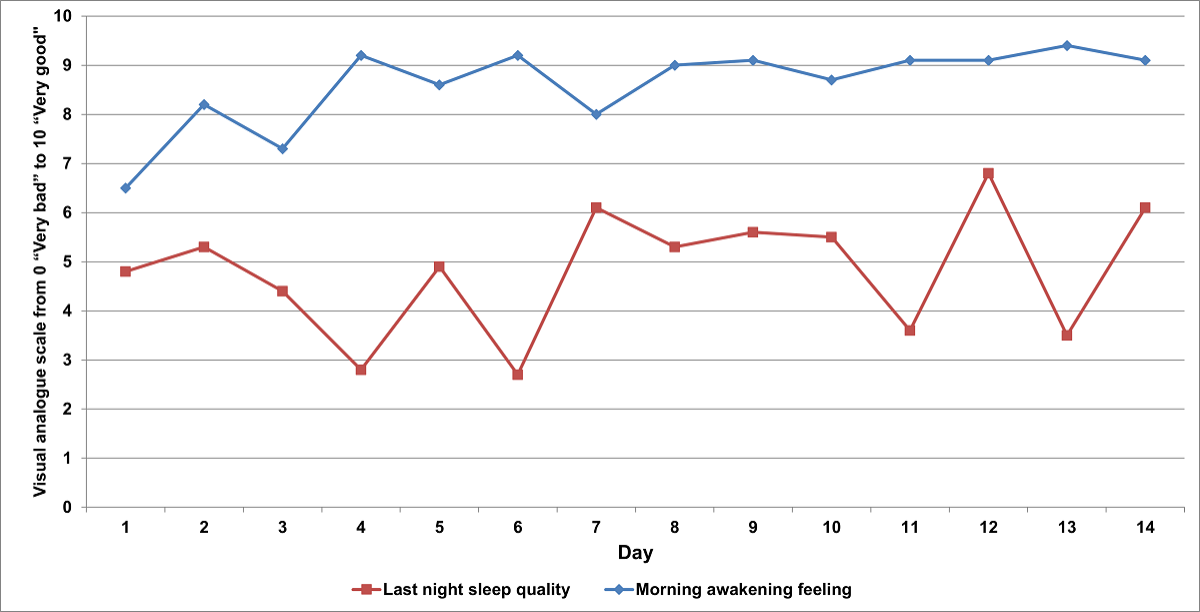
Answers to “Overall, I slept...” (line with squares) and “This morning, upon awakening, I feel...” (line with diamonds) collected from the sleep diary for a single patient.

Finally, a 24-question sleep diary was completed once a day during any of the 4 daily sessions. If the patient did not complete the sleep diary during the after medications session, a prompt would appear at each of the following sessions on the same day. The sleep diary includes a characterization of day-to-day sleep habits; use of stimulating or sleep-promoting beverages, substances, and drugs; and PD-specific or nonspecific nocturnal and diurnal sleep-related symptoms. An example of sleep diary data is shown in Figure 4. Only fully completed tasks (subjective scales, Fit Test, and sleep diary) are recorded and stored by SleepFit.

### SleepFit System

The purpose of SleepFit is twofold: to provide PD patients with a handy home-based system (SleepFit tablet application) suitable for completing the clinical tasks described above (subjective scales, Fit Test, and sleep diary) and provide physicians and researchers with a tool (SleepFit Researcher Portal) to access and consult the data recorded by the application to integrate them with other data sources in the framework of clinical research projects and retrieve them all easily at any time. The modular structure of the SleepFit system enables easy integration of new clinical tasks, functionalities, and external sensors. SleepFit is meant to be shared with other research groups upon mutual agreement.

### SleepFit Application and Patient Interface

SleepFit user interface was developed with an iterative and user-centric strategy to take into account the ergonomic issues specific to this population. We opted for a physical keyboard instead of using the in-application touchscreen keyboard since the motor task was validated on a physical keyboard as well. In addition, the following aspects were also considered in favor of the external keyboard: (1) patients included in our study are mainly elderly subjects who may be more familiar with physical keyboards rather than touchscreens; (2) touchscreen keyboards may raise issues related to skin conductivity (eg, sweaty hands) that could prevent correct key touches from being recorded; and (3) patients may experience a better tactile or sensory feedback and more confidence when dealing with physical buttons. Three versions of the application have been released and tested on patients: alpha, v1.0, and v2.0. The relevant differences and enhancements between versions are described in Table 1.

**Table 1:** Feature improvements between main versions of SleepFit.

Features	Alpha version	Version 1.0	Version 2.0
Graphics	Static background, plain graphics	Dynamic contextual pictograms and wallpapers, session, and task progress bar	Same as v1.0 + font size adapted to the content
Ergonomics	Numerical answers inserted by virtual keyboard	One question per page layout, large-size buttons, predefined numerical answers, +/– buttons for numerical entries	Same as v1.0
Navigation	Session selection relying on the patient	Automatic presentation of sessions, customizable texts and default values, reminders	Same as v1.0 + kiosk mode, multiple language support
Data storage	Remote database (internet connection required)	Store and forward (local + remote database)	Same as v1.0
Remote control	Direct access to remote database	Built-in web application for data querying	Same as v1.0

The alpha version was conceived as a basic software application for tablets bringing together several *ad hoc* tests, scales, and questionnaires employed in clinical routine. Ideas on how to implement the application from the alpha version to v1.0 and subsequently v2.0 arose after direct clinical observation of patients interacting with the application during initial and follow-up visits, comments we received from the participants, and analysis of the more common mistakes they tended to make while using the application interface.

From the prototype (alpha) version to v1.0, the main improvements involved graphics, ergonomics, and navigation. While the layout of alpha version was similar to a paper questionnaire, with plain background, multiple questions on the same page, and standard navigational buttons, graphical and ergonomic improvements in v1.0 included (1) larger, well separated, and accurately positioned buttons to avoid common keypress errors by the participants; (2) ad hoc contextual pictograms and wallpapers; (3) 1 question per page layout; (4) customizable texts, questions, and reminders; and (5) avoidance of open-text fields, which often cause PD patients some difficulty. Regarding navigation, selection of the session to be performed at given times of the day relied on patient choice in the alpha version. From v1.0, a workflow setup guided the patient along the 4 daily sessions by subsequently proposing the correct task to be performed. Additionally, as of v1.0, SleepFit included a log feature, making it possible to record the patient’s in-application behavior (clicks on buttons, touches with corresponding x and y screen coordinates, and timestamp of each action taken by the patient). Furthermore, SleepFit v1.0 was equipped with a local database, which ensured that data was saved even in the absence of an internet connection.

Several questions were added to each session of the subjective scales of v1.0 and retained in v2.0 in order to better describe clinically meaningful features that might influence momentary motor performance or its perception such as mood and fatigue, based on clinical observation and patient feedback. This resulted in increased workload for the patients from alpha to v1.0 and v2.0.

**Figure 5:**
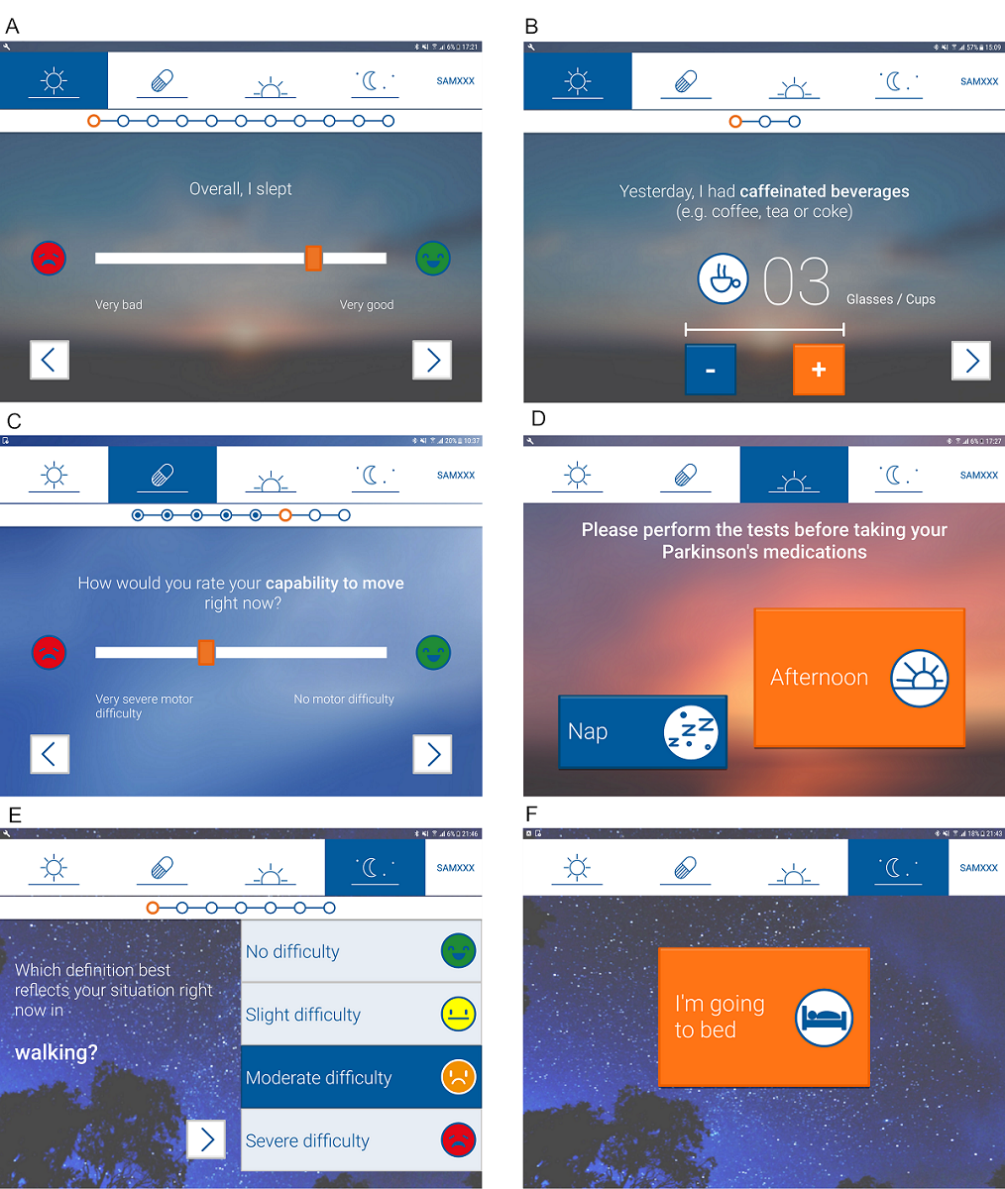
Screenshots of the SleepFit app: A and B) example of sleep diary questions during “on waking” session; C) execution of subjective scales task during “after medications” session; D) choosing between “afternoon” session and performing tasks before taking medication (“nap” session can be enabled by clicking nap button); E) execution of subjective scales task during “evening session”; and F) bedtime recording during "evening" session (patient clicks “I’m going to bed” just before retiring).

Based on patient experience and feedback using SleepFit v1.0, further implementations were included in the September 2018 release of v2.0. In addition to a fine-tuning of ergonomics, the main upgrade to v2.0 was the so-called kiosk-mode feature, which restricts the use of the tablet to the SleepFit application, thus preventing users from accidentally exiting the application and hypothetically interrupting the study. Examples of the SleepFit patient interface are shown in Figure 5.

### SleepFit Researcher Portal

The SleepFit Researcher Portal was conceived and implemented in v1.0 to retrieve and download the data acquired from patients using SleepFit. This portal automatically organizes each patient’s data in sessions and tasks, enabling remote viewing of data from multiple patients. Via the portal, researchers can monitor patient compliance in real time, apply different custom filters (patient ID, date, daily session, etc) to call up specific data, and download data in comma-separated values (CSV) format.

In v2.0, the Researcher portal was further developed as a handy all-in-one tool for data management to be employed in clinical studies using SleepFit. The portal now allows automatic synchronizing and integrating in the study database of the data collected in the case report form generated by the Research Electronic Data Capture application [31,32], uploading of data in CSV format from other sources or devices, or performing intermediate analyses.

**Figure 6:**
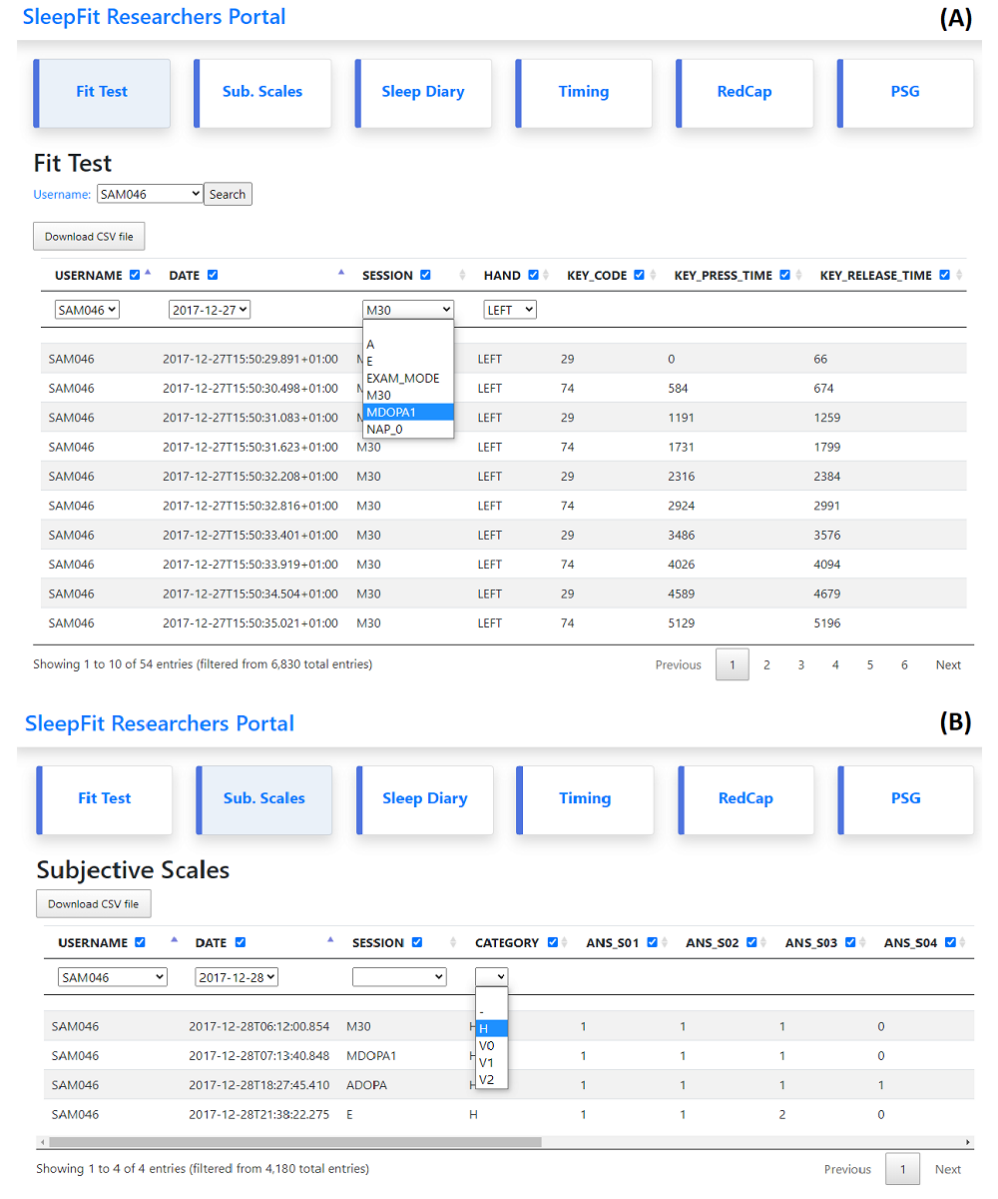
Overview of the SleepFit Researcher Portal for an individual patient: A) Fit Test and B) subjective scales.

A screenshot of the portal is presented in Figure 6. Each button on the action bar opens a different window to consult, filter, or download data from different sources and of different types: Fit Test, subjective scales, sleep diary, timestamps of bedtime and wake times of the 14 days at home, electronic case report form, and intermediate analyses from video-polysomnographic recordings. The researcher can easily filter the data by selecting the patient ID, date, session, and the hand the test was performed with. The filters patient username, date, session, role, and data categorization (H=data collected at home, V0=data collected at the initial visit; V1=data collected at the first follow-up visit) can be applied, and filtered data can be downloaded in CSV format.

### Participant Workload

The study was designed in line with the main objective of the Sleep & Move study, which was to systematically characterize potential spontaneous variations in mobility in relation to sleep. We considered that 9 tasks grouped into 4 sessions per day would achieve a good compromise between acceptable patient workload and sufficiently detailed characterization of mobility and sleep symptoms over a 24-hour period. Each patient was therefore asked to complete 9 tasks per day for 14 days: Fit Test (4 times per day), subjective scales (4 times per day), and sleep diary (once per day).

These 9 tasks were split into the 4 daily sessions according to the Sleep & Move study protocol. Patients executed the tasks at specific times during the day. If a patient did not complete a specific session task within a certain maximum time (which varied based on the session itself), the session expired, and that task could not be performed anymore during that day. In the context of the subjective scales and sleep diary, the alpha version proposed 49 and v1.0 and v2.0 proposed 68 questions per day to each patient.

We included in the analyses the tasks performed from the “evening session” of the first day to the “after medications” session of the 14th day. By the end of the home evaluation period, each patient was expected to have completed 124 tasks (55 subjective scales, 55 Fit Test, 14 sleep diaries), answering a total of 680 questions (alpha version) or 941 questions (v1.0 and v2.0). On-demand nap sessions (performed only if the patients had napped and activated the session request themselves) were not included in the analyses as they could not be scheduled a *priori*.

### Statistical Analysis

Statistical analyses were performed using the R statistics package (R Foundation for Statistical Computing) [33] and Python programming language (Python Software Foundation) [34]. Patient compliance was calculated on the total of the participants and for each of the 3 versions of SleepFit. Two additional analyses were performed on a subgroup of patients having used v1.0 and v2.0.

#### Demographic and Clinical Characteristics

To investigate possible differences in the demographic and clinical characteristics of groups of patients having used different versions of SleepFit (between alpha and v1.0 and between v1.0 and v2.0), we assumed samples coming from a normal distribution and used a *t* test for unequal variances for numeric variables and a proportion test for percentages.

#### Compliance

To assess patient compliance, we computed the ratio of the total number of tasks completed by each patient to the total number of tasks proposed by the application during the home period, as detailed above. The compliance rate is expressed in percentage. The improvement from alpha to v1.0 and v2.0 is evaluated with a classical 1-tailed proportion test.

#### Familiarity With SleepFit Interface

Patient familiarity with the application was computed by analyzing behavior during application use and keeping track of all user interactions with the touchscreen of the tablet, independently of whether the patient clicks on a zone of the screen associated with a command or not. To do this, we compared the number of hit targets (ie, clicks on the parts of the screen where buttons are located) with the total number of touches on the screen. The total screen touches were tracked thanks to the log feature included as of v1.0 of SleepFit. The target ratio, calculated as the ratio between these 2 values, provides an estimate of how accurate the patients were during application use and how well the user interface has been designed. For average target ratio calculation, we excluded the evening session of the first day and the 2 morning sessions (on waking and after medications) of the 14th day because we included only fully completed days in this analysis. The average target ratio was calculated for each patient and further averaged among all patients in order to provide an overall accuracy value.

#### Satisfaction

At the end of each subject’s participation, a survey regarding patient satisfaction with the home-based study using SleepFit v1.0 and v2.0 was administered (see Multimedia Appendix 1). In this analysis we included 7 questions. Three questions focused on user-friendliness of the application: (1) “Did you encounter difficulties using the SleepFit application?” (2) “Did you encounter difficulties in understanding what to do in the different situations proposed?” (3) “Did you encounter difficulties when inputting the answers to the questions proposed (button selection, timing and quantities, sliders)?” Three other questions assessed the graphical interface of the application: (1) “Were texts clearly legible?” (2) “Were the answer and navigation buttons clear?” (3) “Were wallpapers, pictograms, and colors useful for understanding what to do in each different situation proposed?” One final general question assessed the patient’s potential willingness to use the SleepFit application again in the future: “Would you like to use the SleepFit application again in the future if your neurologist proposed it for your clinical follow-up?” All answers were categorical, ranging from 1 (low satisfaction) to 4 (high satisfaction).

## Results

### Participants

**Figure 7:**
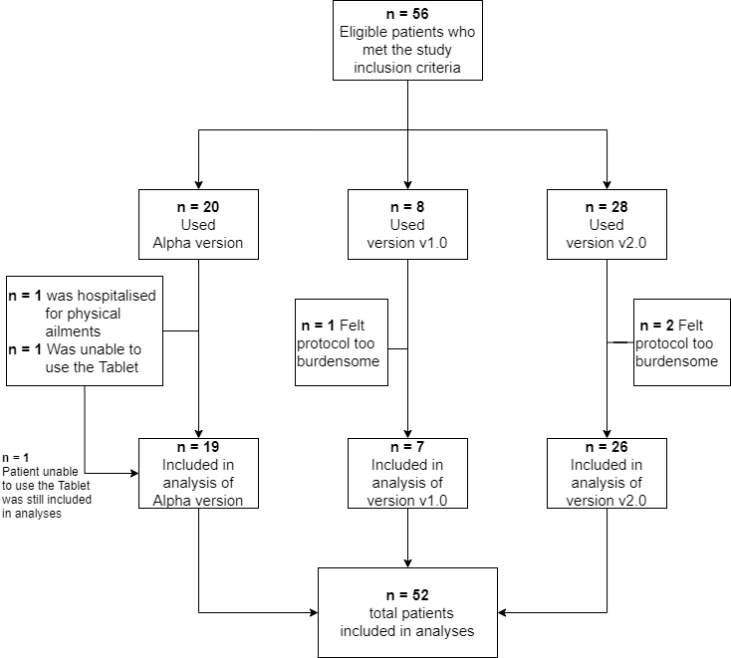
Participant flow.

Of the 56 patients included, 51 completed all study procedures; 1 patient dropped out on day 2 because of an inability to use the tablet (alpha version of SleepFit) and 4 patients prematurely terminated their participation because they perceived an excessive burden due to the study protocol (n=3) or because of physical ailments (n=1). Analyses were conducted on 52 patients (14 females), corresponding to 92.9% of the initial population. The first 19 participants tested the alpha version of SleepFit, 7 used v1.0, and 26 used v2.0. Figure 7 depicts the participant flow. Demographic and clinical characteristics of the study population are shown in Table 2.

**Table 2:** Patient demographic and clinical characteristics for each version of SleepFit and all versions together.

Demographics	Alpha version (n=19)	Version 1.0 (n=7)	Version 2.0 (n=26)	All versions (n=52)
Age (years), mean (SD)	61.9 (9.5)	68.9 (11.5)	66.7 (9.8)	67.8 (9.8)
Sex (female), n (%)	3 (16)	1 (14)	10 (39)	14(27)
Active worker, n (%)	3 (16)	2 (29)	8 (31)	13 (25)
Smartphone user, n (%)	12 (63)	6 (86)	22 (85)	40 (77)
Attempts to independently use SleepFit, mean (SD)	2.0 (1.3)	1.4 (0.5)	1.5 (0.5)	1.7 (0.9)
Parkinson disease duration (years)	5.7 (3.1)	7.8 (7.8)	7.9 (6.5)	7.1 (5.7)
Hoehn & Yahr stage	2.1 (0.2)	1.8 (0.4)	1.9 (0.5)	2.0 (0.4)
MDS-UPDRS^a^ I	8.2 (3.6)	10.6 (5.0)	8.9 (4.8)	8.9 (4.4)
MDS-UPDRS II and IV	10.2 (5.7)	13.7 (8.2)	12.3 (7.7)	11.7 (7.1)
MDS-UPDRS III	37.3 (12.7)	26.1 (9.9)	27.3 (12.4)	30.8 (13.0)
Levodopa daily equivalent dose (mg)	507.7 (271.0)	561.5 (476.9)	666.2 (447.4)	594.2 (395.4)
CIRS-G^b^ total score	8.7 (4.2)	5.4 (4.2)	6.5 (4.2)	7.0 (4.3)
Pittsburgh Sleep Quality Index	6.1 (3.1)	6.4 (3.2)	6.8 (2.8)	6.5 (2.9)
Fatigue Severity Scale	3.9 (1.4)	5.0 (1.7)	4.3 (1.5)	4.3 (1.5)

^a^MDS-UPDRS: Movement Disorder Society–Sponsored Revision of the Unified Parkinson's Disease Rating Scale.

^b^CIRS-G: Cumulative Illness Rating Scale–Geriatric.

### General Design and Development Principles

The implementation of SleepFit brought us to some general considerations which might be helpful for the conception and development of software applications dedicated to patients with PD:

- Tablet format seems to be particularly suitable for software applications to be used by patients with PD
- When different sessions are required to be performed during the day, the software should guide patients through them automatically to avoid mistakes
- Presenting 1 question per page helps patients focusing their attention on the question being asked
- Ergonomics should be considered carefully: large-size buttons, predefined answers, avoiding free-texts answers, simplified navigation to improve usability
- When researchers or clinicians directly provide hardware support, kiosk-mode results are particularly advantageous for those patients not familiar with information technology
- Store-and-forward technology to a cloud-based database helps minimize data loss
- A web platform for data querying allows the investigators/clinicians to provide the patients with remote assistance during the ongoing data collection
- A tool to integrate, synchronize, and retrieve all study data, such as the SleepFit Researcher Portal, might be very useful for data management
- In order to meet the needs of physicians, the structure of the system should be modular and customizable, allowing easy integration of new clinical tests, functionalities, or external sensors

### Demographic and Clinical Characteristics

Demographic and clinical characteristics of each group of patients using different versions of SleepFit are summarized in Table 2. No significant difference among groups was found except for PD duration, which showed a difference between v1.0 and v2.0 (*P*=.03); we interpret this difference to be due to the small size of the sample.

### Compliance

The total expected number of tasks of all patients taken together was 6420. Our analysis revealed that 88.89% (5707/6420) were effectively completed. Detailed estimates of compliance for each version of SleepFit are reported in Table 3.

Considering all 9 daily tasks together, the average daily workload for each patient was 8 minutes and 41 seconds for the alpha version of SleepFit, and 11 minutes and 15 seconds for v1.0 and v2.0. The last 2 versions of the application required more time because the number of questions on the subjective scale task increased from 5 (alpha) to 11 (v1.0 and v2.0) to assess additional motor and sleep-related symptoms. The average total time spent by each patient performing study procedures over the duration of the study was 2 hours and 35 minutes.

**Table 3:** Patient compliance for each version of SleepFit and all versions together. Compliance is reported as the percentage of the tasks completed by the patients over the total of proposed tasks.

Characteristic	Alpha version (n=19) 680 questions in 14 days: 8 mins per day				Version 1.0 (n=7) 941 questions in 14 days: 11 mins per day				Version 2.0 (n=26) 941 questions in 14 days: 11 mins per day				All versions (n=52)		
	Subscale (%)	Fit test (%)	Sleep diary (%)	Subscale (%)		Fit test (%)	Sleep diary (%)	Subscale (%)		Fit test (%)	Sleep diary (%)	Subscale (%)		Fit test (%)	Sleep diary (%)
On waking	88.7	90.6	—^a^	88.8		93.9	—	88.2		89.6	—	88.5		90.5	—
After medications	87.2	86.1	—	88.1		89.3	—	86		86.8	—	86.7		86.8	—
Afternoon	87.5	86.2	—	73.6		82.4	—	92.9		92.9	—	88.3		89.1	—
Evening	86.8	—	—	87.8		88.8	—	86.3		87.4	—	86.7		86.3	—
Total per test	87.6	86.7	93.6	84.6		88.7	96.7	88.3		89.1	99.7	87.5		88.2	97.1

^a^Not applicable.

### Familiarity With SleepFit Interface

**Figure 8:**
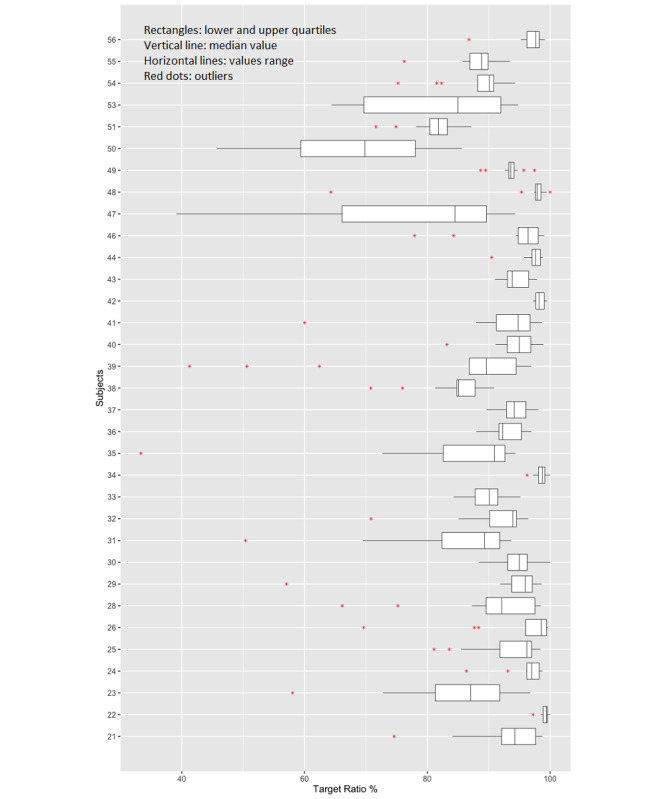
Average target ratio over the home period for the 33 subjects who used SleepFit v1.0 and v2.0.

The first subscale analysis concerning patient behavior during application use was performed on the 33 patients who used v1.0 and v2.0 of SleepFit (ie, the versions equipped with the log feature). This analysis revealed that, on average, the target ratio across all patients was 90.1 (SD 10.4; range 33.3-100). The average target ratio during the home evaluation period for each subject is shown in Figure 8.

### Satisfaction

According to the satisfaction questionnaires (completed by the 26 patients who used v2.0 of SleepFit only), 96% (25/26) would use SleepFit again for clinical purposes for the same period (17/26, 65%) or even for longer (8/26, 31%). Only one patient declared that he would not use it again. For what concerns the usability aspect, 71% (55/78) declared no difficulty, 24% (19/78) slight difficulty, 4% (3/78) moderate difficulty, and 1% (1/78) high difficulty. Finally, the patients rated the graphical interface as follows: 92% (72/78) optimal, 7% (5/78) good, and 1% (1/78) could be better; no one rated the interface very poor.

## Discussion

### Principal Findings

To the best of our knowledge, SleepFit is the first tablet application and remote monitoring system specially designed to be employed in real-life settings for patients with PD that collects subjective and objective clinical data on motor and nonmotor symptoms and subjective sleep information. SleepFit went through several phases of development before reaching v2.0. This innovative, iterative, and user-centric approach targeted to patients with PD is the result of a very close and fruitful collaboration among clinicians, patients, information technologists, and biomedical and telecommunication engineers.

A home-based system could be perceived as intrusive by patients with PD in their daily routines and, thus, may not always be well accepted. Analyzing patient compliance provides a measure of how well these systems are accepted and integrated into the everyday life of patients. We assume this provides an estimate of expected compliance in clinical practice and research too. Compared with the data presented in the literature [35], SleepFit represents a valid alternative to pen-and-paper questionnaires. In fact, it shows higher overall compliance. This result is all the more noteworthy considering that, compared with the study by Stone et al [35], our population includes patients with PD and older age individuals who may experience greater difficulties in dealing with technological devices compared with younger, healthy people. Moreover, the workload required for patients in our study is somewhat greater than in previous studies to date [14-16,20], of which, however, only one evaluated compliance and satisfaction with the system when used on a strictly scheduled protocol [14]. Indeed, our patients answered 68 questions and performed 4 Fit Test tasks every day, for a total of 941 questions and 55 Fit Test tasks by the end of the 14th day. Patients were also asked to respect the time constraints, which could have made it harder for them to adhere to the study since they performed the 4 daily sessions at specific times of the day and had a maximum time allowed for the execution of each task. These aspects may have negatively influenced patient compliance. In spite of this, we observed higher compliance on similar workloads and time constraints compared with studies that used similar technological tools [16].

According to our analysis, there was a significant (*P*=.04) increase of about 2% in compliance between patients who used v1.0 and v2.0 of SleepFit. The sleep diary was completed with high compliance, at 99.7%, in SleepFit v2.0. This value may appear extremely high at first glance but is actually plausible and accurate. The reason is that the sleep diary questionnaire was proposed to the patient at each session throughout the day until it was completed. This means that there were no time constraints on completing the sleep diary, and therefore, better compliance is expected. Conversely, the decrease in compliance during the day from the first morning session to the evening session was not negligible. This may suggest that incremental fatigue accumulating during the daytime may have negatively influenced patient motivation to perform the tasks.

The data collected from the satisfaction questionnaires also provided useful information about patient acceptance of the SleepFit application. Patient willingness to use SleepFit again seems to indicate that this tool might be suitable for clinical follow-up of patients and also for research studies requiring longer participation periods. In fact, almost 31% of the patients who completed the survey reported that they would be willing to use SleepFit again, even for a longer period. The target ratio analysis performed on 33 subjects provided a more objective measure of the usability of SleepFit. We found that the patients were satisfactorily accurate when using the application, with more than 90% of screen touches done on target locations of the screen. This suggests that the design of the graphical interface of SleepFit is suitable for patients with impaired movement capabilities and tremor issues.

### Limitations

We did not distinguish in our analysis if noncompleted tasks were due to technical bugs or a true lack of compliance by the patient. Therefore, our analysis may have underestimated actual compliance, since all missing data were treated as not provided by the patients. Finally, a further limitation of this study is that the conditions in which the tests were performed at home were not verified. However, this limitation is intrinsic in all home-based assessment methods and could only be overcome by means of an increased patient burden (eg, if a researcher came to the patients’ homes during each test session), which would seriously hamper the utility of a home-based approach.

### Future Implementations

Future developments of SleepFit will include data quality checks based on the use of synchronized accelerometric data from wearable sensors. We also foresee the possibility of integrating other tasks and metrics such as electronic tests to assess cognition or other subjective scales exploring motor or nonmotor symptoms of PD. Due to the modular structure of SleepFit, adding new tests or scales is straightforward. SleepFit can thus be easily adapted to different research protocols and for clinical use. The results of our study provide valuable information on the possibility of using SleepFit in other contexts. For instance, a 2-week study paradigm including tests and assessments performed 4 times a day appears to be well accepted by PD patients. SleepFit might also be suitable for clinical follow-up of patients living in remote areas, for chronic patients during the confinement imposed by health authorities in response to the COVID-19 pandemic, or for people with mild cognitive dysfunction, thanks to its user-friendliness.

### Conclusion

SleepFit is an easy-to-use tool that can accurately collect subjective and objective data from patients with PD. It was developed and improved with an iterative user-centric approach. From the lessons learned in this process, essential suggestions emerged for future software application development tailored to PD patients. Although the use of SleepFit should be further tested in larger populations, both for clinical follow-up and in other home-based research studies, this application proved to be a very promising tool to increase patient compliance and assist researchers in surveying patients during data collection and data management.

## Appendix

Multimedia Appendix 1 Satisfaction questionnaire for the SleepFit app.

## Abbreviations

CSV: comma-separated values
PD: Parkinson disease
