# Supplementary material for: The SleepFit Tablet Application for Home-Based Clinical Data Collection in Parkinson Disease: User-Centric Development and Usability Study
Source: JMIR Mhealth Uhealth. 2021 Jun 8;9(6):e16304. doi: 10.2196/16304 (PMC8262669; doi:10.2196/16304)
Supplement: Multimedia Appendix 1 [file mhealth_v9i6e16304_app1.docx]

The following questions are meant to evaluate your experience of using the *Sleep Fit application in the* last 14 days. For each question, choose only one answer

***SECTION A:***

***The following questions refer to the ease of use of the application.***

***1) Understanding:***

**a)** The explanation for how to use the application by researchers was: (1pt)

1) ⬜ Very clear

2) ⬜ Pretty clear

3) ⬜ Unclear

4) ⬜ Unclear

**b)** Have you had difficulty learning how to use the application? (1 pt)

1) ⬜ No difficulties

2) ⬜ Slight difficulty

3) ⬜ Moderate difficulty

4) ⬜ High difficulty

**(c)** Have you had difficulty understanding the content of the applications proposed by the application?

(1 pt)

1) ⬜ No difficulties

2) ⬜ Slight difficulty

3) ⬜ Moderate difficulty

4) ⬜ High difficulty

**(d)** Have you had difficulty understanding what to do in the various situations proposed? (1 pt)

1) ⬜ No difficulties

2) ⬜ Slight difficulty

3) ⬜ Moderate difficulty

4) ⬜ High difficulty

***2) Use:***

**a)** Have you had difficulty selecting the answers (buttons, times and quantities, scrolling of the

bars)? (1 pt)

1) ⬜ No difficulties

2) ⬜ Slight difficulty

3) ⬜ Moderate difficulty

4) ⬜ High difficulty

**b)** Have you had any problems switching between different application screens? (1 pt)

1) ⬜ None

2) ⬜ Few

3) ⬜ Enough

4) ⬜ Many

**c)** Have you had difficulty using the external keyboard? (1 pt)

1) ⬜ No difficulties

2) ⬜ Slight difficulty

3) ⬜ Moderate difficulty

4) ⬜ High difficulty

***SECTION B:***

***The following questions refer to your level of application liking.***

***1) Graphics:***

**a)** Was the size of the texts legible? (1 pt)

1) ⬜ Yes, well readable

2) ⬜ Quite legible

3) ⬜ Unleatable

4) ⬜ No, poorly readable

**b)** Were the response keys and progress keys between screens clear? (1 pt)

1) ⬜ Yes

2) ⬜ Pretty clear

3) ⬜ Few clear

4) ⬜ No

**c)** Backgrounds, figures, colors, and images have been helpful in understanding what to do in the

different situations proposed? (1 pt)

1) ⬜ A lot

2) ⬜ Enough

3) ⬜ Little

4) ⬜ No

**d)** Were the backgrounds, figures, colors, and images to your liking? (1 pt)

1) ⬜ A lot

2) ⬜ Enough

3) ⬜ Little

4) ⬜ No

***2) Acceptability:***

**a)** Have you had a hard time using a tablet? (1 pt)

1) ⬜ No difficulties

2) ⬜ Slight difficulty

3) ⬜ Moderate difficulty

4) ⬜ High difficulty

**b)** Has the commitment to perform the tests affected the performance of your daily activities?

(1 pt)

1) ⬜ No

2) ⬜ Little

3) ⬜ Enough

4) ⬜ A lot

**c)** Did you have difficulty running the tests at exactly the time of day required?

(1 pt)

1) ⬜ No

2) ⬜ Little

3) ⬜ Enough

4) ⬜ A lot

**d)** Have you forgotten to hold some sessions? (1 pt)

1) ⬜ Never

2) ⬜ Rarely

3) ⬜ Sometimes

4) ⬜ Often

**e)** How do you judge the amount of sessions on each day? (1 pt)

1) ⬜ Well acceptable

2) ⬜ Quite acceptable

3) ⬜ A bit high

4) ⬜ Too high

**f)** How do you judge the amount of questions in each session? (1 pt)

1) ⬜ Well acceptable

2) ⬜ Quite acceptable

3) ⬜ A bit high

4) ⬜ Too high

**g)** Do you think that having participated in the study has changed your habits? (1 pt)

1) ⬜ Yes, for the better: my habits have become more regular

2) ⬜ No

3) ⬜ I do not know

4) ⬜ Yes, for the worse (specify): ________________________________________

***SECTION C:***

***The following questions refer to the perceived utility using the application..***

**a)** Considers that this application is adequate to describe your sleep and

drowsiness in the day? (1 pt)

1) ⬜ Very adequate

2) ⬜ Quite adequate

3) ⬜ Inadequate

4) ⬜ No

**b)** Considers that this application is adequate to describe its ability to move

in the day? (1 pt)

1) ⬜ Very adequate

2) ⬜ Quite adequate

3) ⬜ Inadequate

4) ⬜ No

**c)** Do you think this application is adequate to describe your emotions in the day?

(1 pt)

1) ⬜ Very adequate

2) ⬜ Quite adequate

3) ⬜ Inadequate

4) ⬜ No

**(d)** Considers that this application has been useful in better recognising certain aspects of the

his Parkinson's disease? (1 pt)

1) ⬜ Yes

2) ⬜ You probably

3) ⬜ Probably not

4) ⬜ No

**c)** Would you participate in other research using this application in the future? (1 pt)

1) ⬜ Yes, even for a longer period

2) ⬜ Yes, for the same period (2 weeks)

3) ⬜ Yes, but for a shorter period

4) ⬜ No

**f)** Would you use this application again to be better followed by your neurologist for your Parkinson's disease? (1 pt)

1) ⬜ Yes, even for a longer period

2) ⬜ Yes, for the same period (2 weeks)

3) ⬜ Yes, but for a shorter period

4) ⬜ No

***SECTION D:***

***On a scale of 1 to 10 (cm) indicate on this line its level of application* satisfaction *(0.4 pt):***

**very satisfied not at all satisfied**

**Do you have any other comments, advice, or suggestions?**

________________________________________________________________________________________________________________________________________________________________________________________________________________________________________________________________________________________________________________________________________________________________________________________________________________________________________________________________________________________________________________________________________________________________________________________________________________________________________________________________________________________

**Score:**

**TOTAL: ____/100**
